# CRIS.py: A Versatile and High-throughput Analysis Program for CRISPR-based Genome Editing

**DOI:** 10.1038/s41598-019-40896-w

**Published:** 2019-03-12

**Authors:** Jon P. Connelly, Shondra M. Pruett-Miller

**Affiliations:** 1 St. Jude Children’s Research Hospital, Department of Cell & Molecular Biology, Memphis, 38105 USA; 2 St. Jude Children’s Research Hospital, Center for Advanced Genome Engineering, Memphis, USA

## Abstract

CRISPR-Cas9 technology allows the creation of user-defined genomic modifications in cells and whole organisms. However, quantifying editing rates in pools of cells or identifying correctly edited clones is tedious. Targeted next-generation sequencing provides a high-throughput platform for optimizing editing reagents and identifying correctly modified clones, but the large amount of data produced can be difficult to analyze. Here, we present CRIS.py, a simple and highly versatile python-based program which concurrently analyzes next-generation sequencing data for both knock-out and multiple user-specified knock-in modifications from one or many edited samples. Compared to available NGS analysis programs for CRISPR based-editing, CRIS.py has many advantages: (1) the ability to analyze from one to thousands of samples at once, (2) the capacity to check each sample for multiple sequence modifications, including those induced by base-editors, (3) an output in an easily searchable file format enabling users to quickly sort through and identify correctly targeted clones.

## Introduction

Genome editing using CRISPR-Cas9 has not only made the rapid creation of custom cell and animal models possible but it has also made these reagents an expected component within a wide variety of research fields. CRISPR-Cas9 is a two-component system composed of a Cas9 nuclease and a guide RNA molecule (gRNA). The gRNA directs the Cas9 nuclease to a targeted site within a genome and can be swapped out to redirect the nuclease activity of Cas9. After binding the target site, the Cas9 nuclease creates a double-strand break (DSB) in the genomic DNA. These breaks are typically repaired by one of two general pathways: non-homologous end-joining (NHEJ) or homology-directed repair (HDR). If the break is repaired by the NHEJ pathway, insertions or deletions (indels) may be created leading to gene disruption. Alternatively, if a donor template containing homology to the cut site is introduced into the cell along with the nuclease, the break may be repaired by HDR such that nucleotide substitutions, insertions, or deletions present on the donor will be incorporated into the genome. These targeted modifications can be made in cells or whole animals to generate better models of disease, to test basic scientific hypotheses, and potentially, for therapeutic applications.

In theory, genome editing is remarkably easy, but in practice it requires a great deal of time, knowledge, and ideally, some capital equipment to expedite the process and increase the likelihood of success. Moreover, for most research applications, it is necessary to derive a clonal population of cells containing the modification of interest, which requires considerable time and effort. Obtaining precisely edited clones without using selection markers typically requires screening hundreds of clones.

Commonly used methods for screening genome-edited populations and clones include PCR followed by TOPO cloning and Sanger sequencing, Tracking of Indels by Decomposition (TIDE), and the T7E1 assay^1,2^. These methods provide varying information ranging from the presence of indels (T7E1 and TIDE) to the specific sequence information (PCR/TOPO cloning) of each clone. Furthermore, these methods require the end-user to invest significant time into analyzing each individual clone and make screening hundreds of clones inefficient.

Previously, we compared editing efficiencies reported by targeted next-generation sequencing (NGS) and several other commonly used validation strategies and spotlighted the limitations of each technique^3^. Overall, because targeted NGS analysis provides the sequence identity, the size of indels, and the frequency of indels, many labs would prefer to use this approach. Moreover, as the price of NGS has fallen, it has become more financially accessible for researchers. However, the large amount of data generated through NGS requires efficient and accurate analysis programs. An ideal analysis program for analyzing CRISPR-based editing would feature: 1) the ability to analyze multiple samples at one time, 2) the capacity to search for multiple editing events created by ssODNs or base-editors, 3) run on a local computer and not require uploading potentially sensitive data online, and 4) require minimal setup. Currently, several programs are available to analyze NGS results for different CRISPR-Cas9 induced genome editing outcomes^4–7^. These programs accurately analyze certain aspects of NGS data, but each has specific limitations which reduce the utility of each platform (Table 1).

**Table 1:** Software Programs for analyzing CRISPR activity.

Program	Local	>1 Sample	>1 donor search	Master Summary	Journal
Cas-analyzer	No	No	No	No	*Bioinformatics*(6)
CRISPR-GA	No	No	No	No	*Bioinformatics*(5)
CRISPResso	Yes	No	No	No	*Nat Biotech*(4)
CRISP-DAV	Yes	Yes	No	No	*Bioinformatics*(7)
CRIS.py	Yes	Yes	Yes	Yes	

Here, we introduce CRIS.py, a python-based program that analyzes NGS data obtained from both pools of cells and individual clones in an easy-to-use, high-throughput manner. CRIS.py directly analyzes demultiplexed fastq files produced from an NGS run, searches each fastq file in a directory, and reports read counts, indel sizes, indel frequencies, and sequence identities. This is achieved through a text-based search algorithm. CRIS.py allows users to measure frequencies of different types of edits, with all known platforms of editors in a multiplexed fashion and distinguishes true editing events from background sequencing and PCR amplification errors.

## Material and Methods

**Figure 1:**
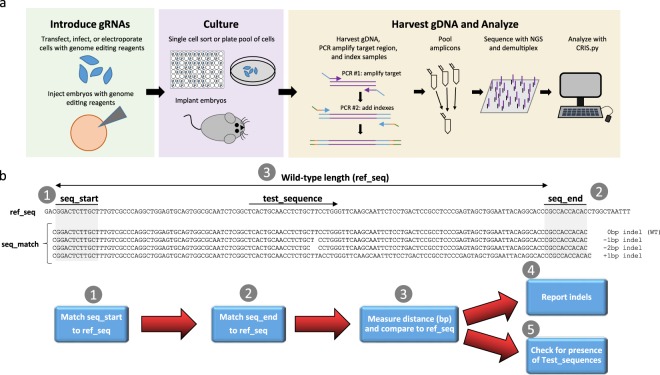
Schema of CRIS.py screening method. (**a**) Workflow for generating and analyzing genome edited cells and mice. Genome editing reagents are introduced into desired cell type/organism. The cells or embryos are cultured and given time for the editing to occur. Genomic DNA (gDNA) is harvested, target regions amplified and indexed, amplicons pooled and sequenced by NGS. In the final step, sequencing results are analyzed with CRIS.py. (**b**) Flow chart to analyze NGS results using CRIS.py. Two short user-defined sequences (seq_start and seq_end) are matched to the specified wild-type amplicon sequence (ref_seq) and the total length of characters between the sequences is measured to calculate the wild-type length. (1 and 2) Every sequence in each fastq file is checked for seq_start and seq_end (a match is termed a seq_match), and (3) the length is measured and compared to the reference. (4) all lengths in a fastq file are quantified and reported. (5) Each seq_match is checked for user-defined test_sequences.

CRIS.py is a simple text-based search and analysis program which utilizes Python 2.7 and the pandas module. Prior to CRIS.py analysis, users must first generate CRISPR modified cells or organisms (Fig. 1a). This is typically done by delivering Cas9 and gene specific gRNAs into cells or embryos. After culturing or birth of pups, genomic DNA is harvested and the target site amplified and prepped for NGS allowing hundreds to thousands of amplicons to be sequenced in parallel. Finally, NGS results are demultiplexed and then analyzed using CRIS.py. In the CRIS.py script, users are able to directly make modifications and change variables specific for their experiment (Fig. 1b and Supplemental Fig. 1). These variables include the ‘ID’ which is used to label the output files, a reference sequence (‘ref_seq’), two sequences flanking the gRNA site (‘seq_start’ and ‘seq_end’), and a list of names and sequences that the user wants to query within the samples (‘test_list’). Analysis is strand specific. For example, in the fastq files only the forward (R1) or reverse strand (R2) is reported. Therefore, all user inputted data should match the strand being analyzed. CRIS.py uses inputs to search and find reads within a directory of fastq files. When CRIS.py is run, the two flanking sequences (seq_start and seq_end) are matched to the user-inputted reference amplicon sequence (ref_seq) to determine the normal distance (in base pairs) separating the two sequences and establish a wild-type reference length. Next, all reads in a fastq file containing seq_start and seq_end (termed a seq_match) are counted and used for further analysis. For each seq_match, CRIS.py calculates the distance between the seq_start and seq_end and compares that length to the established wild-type reference length. An amplicon containing no indel and therefore of wildtype length is reported as a 0 bp indel. Amplicons with a shorter or longer length will be reported back with the corresponding indel sizes. Indel frequencies are then calculated by summing the total number of each indel size and dividing by the total number of seq_matches. Additionally, CRIS.py can determine the frequency of one or more user-specified modifications such as an HDR event created using single-stranded oligodeoxynucleotide (ssODN) donor templates or base changes created using CRISPR-Cas base-editors. To achieve this, CRIS.py counts the number of seq_matches that contain the user-specified modification (termed a test_sequence) and divides that number by the total number of seq_matches. Additionally, CRIS.py allows multiple user-specified test_sequences to be queried concurrently. CRIS.py source code, documentation, and all example files are available for download on GitHub (https://github.com/patrickc01/CRIS.py) and are free for non-profit use. Additionally, a detailed tutorial video is available (https://s.stjude.org/video/player.html?videoId=6000021936001).

### Output files

After the fastq files have been analyzed, CRIS.py creates a folder based on the user-inputted ‘ID’ and writes two files– a CSV file and a TXT file. The CSV file is a master summary file which contains the fastq file names, total read counts, frequencies of user-defined test sequences, the top indels found in each file, and quality control checks. This file is easily sortable enabling quick and accurate identification of correctly targeted clones from hundreds or even thousands of input clones. Additional columns have been added to the far right of the CSV file reporting the frequency (without read counts) for each “test_sequence” for ease of use when sorting for desired clones. Additionally, the TXT output file contains the top exact reads for each fastq file and the read counts for each sequence allowing the user to examine, align, or otherwise verify the genotype of a potential clone or animal. Because sequences are binned based on exact matches, it is easy to visually assess which sequence reads are true contributors to the sample genotype and which are results of poor-quality sequencing and/or PCR or sequencing error.

## Results

### Measuring genome editing outcomes in pools of edited cells

**Figure 2:**
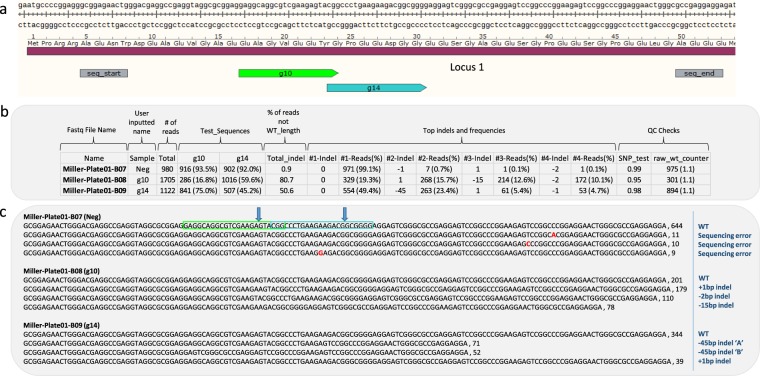
Analysis of NHEJ activity in pools of cells. (**a**) Layout of target locus with gRNAs (g10 and g14), seq_start, and seq_end labeled. (**b**) A representative CRIS.py output CSV file for CRISPR activity in different pools of cells (fastq files). (**c**) A representative CRIS.py TXT output file lists the most common reads found in each fastq file along with the read counts for each read with a comma separating the sequence read and the read count. Note that the top reads for the negative control are all of WT length and only vary by single base pair sequencing errors (red base pairs). Treated wells (g10 and g14) contain a large portion of indels as can easily be seen with the aligned reads of different lengths and corresponding read counts. Blue arrows indicated the respective gRNA cut site.

We have used CRIS.py to analyze the activity of thousands of gRNAs and identify hundreds of single-cell–derived clones. As an example, we show the analysis of two pools of cells each treated with Cas9 and a different gRNA to measure indel frequencies as a read-out for gRNA cleavage activity (Fig. 2a). CRIS.py analysis demonstrates that samples g10 and g14 contain total indel frequencies of 80.7% and 50.6%, respectively (Fig. 2b, column Total_indel). Comparatively, the negative control (Neg) contains less than 1% total indels (Fig. 2b). Indels found in the negative control most likely result from normal slippage of the polymerase during DNA synthesis, either during the initial PCR or during sequencing, and do not align to either gRNA cut site as shown Fig. 2c.

By default, CRIS.py reports the top eight indel sizes by frequency for each sample. For example, the column labeled “#1-Indel” reports the size (in base pairs) of the most frequent indel in a given sample. The column labeled “#1-Reads(%)” reports the total number of reads and the percentage of reads with that indel size. Accordingly, “#2-Indel” and “#2-Reads(%)” columns report the indel size and frequencies of the second most abundant indel and so on. An indel length of 0 bp indicates that no indel was created and the amplicon is of wild-type length compared to that of the reference amplicon. Because NHEJ events can also result in nucleotide transitions or transversions, which would not affect the amplicon length, the gRNA target site can also be used as a test sequence to check for a match to the WT sequence. In the g10 sample, the most-frequent indels present, besides the 0 bp indel, are a +1 bp indel (15.7%) and an in-frame −15 bp indel (12.6%). The g14 sample shows a 0 bp indel followed by a large deletion of 45 bp (23.4%), and a 1 bp insertion (5.4%) (Fig. 2b). Figure 2b is representative of the master summary CSV file output. The read counts and sequences of the most-common reads observed are shown in Fig. 2c and correspond to the data outputted as a TXT file. The negative control shows several different reads; but notably, there is only 1 prominent consensus read, and all reads are the same length. Differences in sequences for the control sample can be attributed to sequencing errors and do not occur at the cut site for either tested gRNA (Fig. 2c, red base pairs). The samples treated with Cas9 and either gRNA g10 or g14 show a variety of read lengths, with indels occurring at each of the respective gRNA cut sites (Fig. 2c). Notably, indels of the same length are binned together in the CSV summary file (Fig. 2b) but can be separated when examining the exact sequence reads reported in the TXT file (Fig. 2c). For example, a −45 bp indel is reported to represent 23.4% of the reads in the g14 sample (Fig. 2b, column #2-Reads(%)). Upon examination of the top sequence reads shown in the TXT file (Fig. 2c), one can see that two distinct −45 bp indels are within the fastq sample. These data demonstrate that NHEJ activity of gRNAs can be accurately measured and reported using CRIS.py.

Knocking out genes in a cell line or animal model aids in studying the overall function of or requirement for a gene. In cases where it is necessary to study protein domains or roles of specific amino acids within a protein (such as modeling patient-specific mutations), knocking out the gene will not suffice. In these instances, a precise point mutation must be created at the target site.

### Measuring homology directed repair (HDR) events in pools of cells

**Figure 3:**
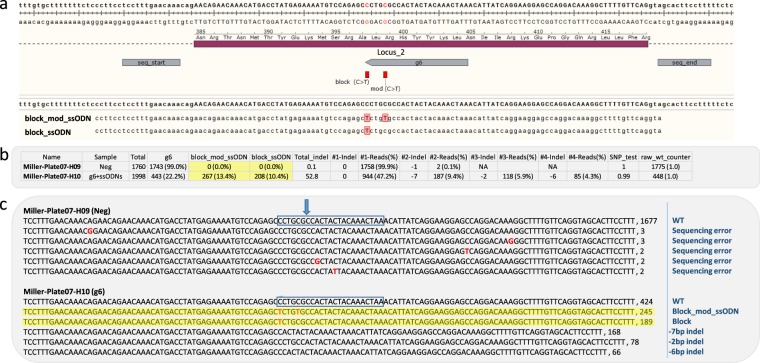
Analysis of CRISPR activity and gene targeting in a pool of cells transfected with gRNA, Cas9, and 2 different ssODNs. (**a**) Layout of targeted genomic region with the gRNA target site, seq_start, and seq_end labeled. Target modifications are shown in red. Two ssODNs (block_mod_ssODN and block_ssODN) used for gene targeting are aligned to WT genomic locus. (**b**) The CRIS.py summary CSV file shows cells treated with Cas9, gRNA, and ssODNs contain a high frequency of indels (52.8%). Additionally, 13.4% and 10.4% of the pool underwent correction with the block_mod_ssODN or the block_ssODN, respectively (yellow highlight). (**c**) CRIS.py TXT file results shows top reads found in each of the 2 pools of cells. Reads resulting from gene targeting by ssODNs highlighted in yellow. Sequencing errors, mod, and block modifications are indicated in red. Blue arrow indicates the gRNA cut site.

CRIS.py can accurately report one or more targeted HDR events. Users may input multiple desired HDR events as test_sequences within the test_list portion of the script (Supplemental Fig. 1b, parameter 6). CRIS.py will search for each test_sequences and report the frequencies of each in the master summary CSV file. As an example, we used CRIS.py to analyze sequences obtained from cells treated with a gRNA, Cas9, and two distinct ssODN donors (Fig. 3a). As shown in Fig. 3b, 99% of the negative control sequences were wild-type for the g6 gRNA site (Fig. 3b, Column g6), and 99.9% had the wild-type amplicon length (Fig. 3b, Column #1-Reads(%)). Besides an occasional transition or transversion at the cut site, the differences between the exact matches to the g6 target site and the length matches to the amplicon can be attributed to differences in sequencing errors for substitutions versus that for insertions or deletions. The overall sequencing error rate for substitutions (per base pair) has been reported to be 0.21% to 0.42%^8^. The overall sequencing error rate for insertions and deletions is ~100X lower than the substitution rate^9^. We, therefore, recommend running a negative control sample in addition to experimental samples to account for context-specific sequencing errors within each amplicon. For quick and easy interpretation, the total amount of indel formation is also reported for both the negative control and treated samples. The “Total_indel” column reports the percentage of reads that do not have the wild-type length (indel size of 0 bp). The negative control sample contains a Total_indel of 0.1%, whereas the treated sample contains 52.8% indels. The negative control sample is reported to contain no ssODN-specified modifications (Fig. 3b, highlighted in yellow). In contrast, 23.8% (13.4% block_mod_ssODN and 10.4% block_ssODN) of the sequences from the cells treated with gRNA, Cas9, and the two ssODN donors contain the desired modifications. Sequence information from the fastq files is reported in the TXT file and allows the user to verify sequence identity or further examine and align indels to a reference (Fig. 3c). These data demonstrate that several HDR events along with gRNA-induced NHEJ events can be concurrently analyzed in the same sample using CRIS.py.

### Measuring genome editing outcomes in single-cell–derived clones

**Figure 4:**
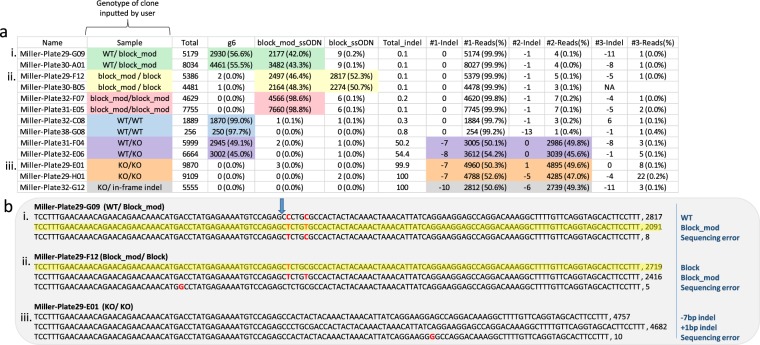
CRIS.py analysis of single-cell-derived clones from a pool of cells treated with gRNA, Cas9, and 2 ssODNs. (**a**) The CRIS.py summary CSV file is easily sortable and allows for quick identification of clones with all combinations of potential modifications. The most relevant columns for each genotype listed are highlighted with corresponding colors. (**b**) CRIS.py summary TXT file showing most common reads found in 3 representative wells/clones (i., ii., iii.) with a mix of WT, modified, and KO alleles. Sequencing errors, mod, and block modifications highlighted in red. Layout of target locus, gRNAs, and ssODNS is the same as shown in Fig. 3. Blue arrow indicates the gRNA cut site.

When creating a knock-out or custom knock-in cell line, a necessary step is screening single-cell–derived clones for the desired modification. To highlight the utility of CRIS.py to screen for modified clones, we sorted a pool of potentially modified cells at one cell per well into four, 96-well plates; harvested gDNA; and sequenced the target site by performing two-step PCR for NGS^10–13^. All clones were analyzed during one program iteration; examples of modified clones are shown in Fig. 4a. Sorting the CSV file by modification quickly and efficiently identifies different modification types. Wild-type clones can be distinguished from homozygously or heterozygously modified clones. Moreover, clones containing out-of-frame knockouts or in-frame indels are also readily apparent. CRIS.py calculates the ratio at which each indel is present within a sample. This also allows the copy number of an allele in a sample to be inferred (Supplemental Fig. 2). The top exact sequence reads found within each sample as reported by the demultiplexed well number (shown in the “Name” column of the CSV file) are also reported to further clarify the exact genotype of a given clone (Fig. 4b). This method allows easy analysis and selection of clones for further experiments.

### Measuring genome editing outcomes of custom edited animals

**Figure 5:**
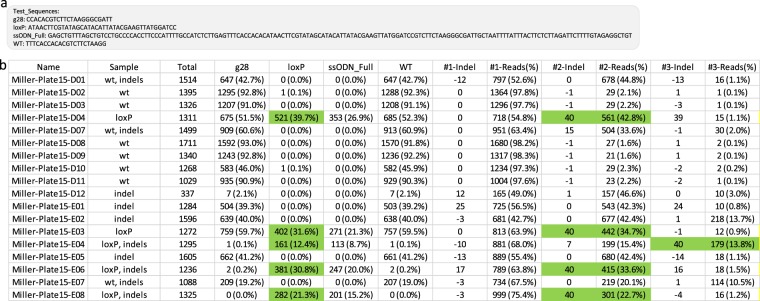
CRIS.py analysis of pups injected with gRNA, Cas9, and loxP containing ssODN. (**a**) Input and Test_sequences for CRIS.py program to analyze pups after direct embryo injection. (**b**) CRISP.py master summary file results from screened pups. LoxP site integration is detected by both the “loxP” test sequence and in the top identified indels as the loxP integration creates a 40 bp insertion (34 bp for LoxP site + BamHI site).

Custom edited animal models can also be created by using the CRISPR-Cas9 platform. This technique is typically achieved by directly injecting gRNA, Cas9, and ssODNs into one-cell stage embryos and transferring embryos to foster mothers. It is worth noting that the resulting animals can potentially be mosaic because the gRNA-induced modification does not always occur at the one-cell stage. Here, we show an example of CRIS.py analysis on several mouse pups resulting from single-cell embryo injections (Fig. 5). Specifically, Cas9 mRNA, gRNA, and an ssODN designed to insert a loxP site along with a 6 bp restriction site into the cut site were injected into single-cell mouse embryos. Tail snips were taken from pups, genomic DNA was harvested, and the gRNA cut site was sequenced to identify animals containing the desired integration. CRIS.py correctly identified pups containing loxP integrations, wild-type animals, and those containing indels at the target site. Pups containing three or more alleles may be inferred to be mosaic (Fig. 5, pups 15-E02, 15-E04). It is not always possible to tell whether pups containing two or fewer modifications are heterozygous or mosaic without further breeding. CRIS.py quantifies the percentage of loxP-containing reads in each pup, such that pups containing the highest percentage of loxP reads can be identified and bred, reducing the amount of breeding required to derive the final mouse line.

### CRIS.py comparison to alternative CRISPR analysis program

To further test the accuracy and utility of CRIS.py, we performed a direct comparison with the popular analysis program CRISPResso^4^. As shown in Table 2, CRIS.py and CRISPResso calculate similar read counts and indel rates when run on samples treated with Cas9 and gRNAs. Both programs also measure similar rates of gene targeting when using a single ssODN. However, when multiple ssODNs are used in an experiment, CRISPResso is unable to correctly parse the targeted modifications resulting from additional donors, and instead aggregates other gene targeting events into the ‘Indels’ column. Conversely, CRIS.py accurately quantifies the additional gene targeting events. This is critical when more than one ssODN donor is introduced into a sample at once, such as when trying to create a heterozygous modification, or when using CRISPR-Cas9 base editors which can result in multiple different combinations of base pair modifications.

**Table 2:** Comparison of CRIS.py to CRISPResso.

Sample	Analysis Program	Total Reads	Unmodified	Indels	HDR1	HDR2
Fig. 2. Pooled NHEJ 1-B08 (g10)	CRISPResso	1586	20.20%	79.80%	—	—
	CRIS.py	1122	16.80%	80.70%	—	—
Fig. 3. Pooled NHEJ with 2 ssODNs 7-H10 (g6)	CRISPResso	2028	21.40%	64.4%	13.30%	Unsupported
	CRIS.py	1998	22.2%	52.8%	13.4%	10.4%
Fig. 4. KO clone 32-G12	CRISPResso	5594	0%	100.00%	—	—
	CRIS.py	5555	0%	100.00%	—	—
Fig. 4. Clone modified with 2 ssODNs 29-F12 (ii)	CRISPResso	5432	0.60%	53.20%	46.20%	Unsupported
	CRIS.py	5386	0.0%	0.1%	46.4%	52.3%

Additionally, CRIS.py allows easy analysis of hundreds to thousands of fastq files compared to CRISPResso, which only allows for analysis of a single sample (clone) at a time. In brief, by incorporating indexes/barcodes into NGS primers, users can pool together large numbers of samples into a single NGS run. When screening clones, primers containing a unique index set can be used in each well of a plate. After sequencing, the reads can be grouped based on index sequence to allow analysis of each well. CRIS.py runs analysis on all fastq files in a directory at once. However, CRISPResso requires manually uploading each set (forward and reverse reads) of fastq files from an NGS run, running the program, and subsequent analysis on each set. For a single 96-well plate, this would require 96 iterations of uploading, running, and analysis. This process is inefficient in situations such as screening hundreds of clones for a desired modification. Furthermore, although CRISPResso outputs visually pleasing graphical data (pie charts, bar graphs, and color-coded sequence alignments), the output is limited in its utility when comparing results from hundreds of samples to quickly find the most desirable clones. In contrast, CRIS.py screens thousands of fastq files in a matter of seconds to minutes and summarizes all results in a CSV file that can be read by any spreadsheet software and which can be sorted and ranked to look for knockouts, homozygous or heterozygous gene targeted clones, or any combination of alleles including modifications made using Cas9-base editors.

To demonstrate the utility of CRIS.py for analyzing sequencing data obtained from samples treated with Cas9-base editors, we ran CRIS.py on six such samples sequenced on one sequencing run (Supplemental Fig. 3a). Multiple test_sequences can be entered to search for desired editing events. In this example, we include all C-to-T changes within the predicted cytosine base-editing window. The same sequencing files were also run using CRISPresso (Supplemental Fig. 3b). CRISPresso was not able to specifically quantify desired base editing events, but the percentages of each editing event can be determined using the sequence-alignment outputs (one alignment for each sample). Overall, base-editing efficiencies were highly congruent when analyzed with CRIS.py or CRISPresso (Supplemental Fig. 3c).

### Quality control measures

Because CRIS.py relies on an exact sequence match between the user-entered sequences and the NGS reads, a series of quality control checks are performed. For example, a flanking sequence (seq_start or seq_end) will not align to the fastq reads if the genome being sequenced contains a SNP within that flanking sequence. To identify potential problematic SNPs within one of the flanking sequences, a ratio is calculated between the total number of times the seq_start and seq_stop sequences are counted in each fastq file (Supplemental Fig. 4). In a sample with no SNPs, the ratios should be roughly equal (1:1). However, if a SNP exists in the seq_start sequence on one allele but not in the seq_stop sequence, then the upstream sequence will only be matched at half the frequency of the downstream sequence giving a ratio of 1:2 (or 0.5). This is reported as the “SNP_test” in the CSV master summary file and allows a quick, simple data check for missing reads. The SNP_test will also be affected if a large indel results in the loss of either the seq_start or seq_end. Additionally, SNPs in the genome could also exist at both flanking sequences for one or more alleles, which would result in a roughly equal number of reads aligning to the reference sequence for both flanking sequences and omitting all reads that contain the SNPs. To test for this potential outcome, a ratio is calculated between the number of reads found for the first test sequence that are found within the raw fastq file and the number of reads for the same sequence that are found in the seq_match sequences. This is reported as the “raw_wt_test” (Supplemental Fig. 5). It should be noted that this counter will equal zero for total knockout clones if the first test sequence is the gRNA that was used for the knock-out modification.

## Discussion

Herewithin, we detail a versatile, high-throughput NGS pipeline for analyzing genome editing outcomes. Although NGS generates enormous amounts of sequencing data in just hours to days, several limitations are inherent to using NGS, and specifically, targeted NGS. The largest limitation is read length. The most common NGS sequencers, the MiSeq and Ion Torrent, can analyze read lengths up to 600 bp or 400 bp, respectively. Additionally, if a large deletion occurs that abolishes a PCR primer binding site, the allele will not be amplified or sequenced. Moreover, another inherent limitation in using targeted NGS data to determine indel or knock-in mutations is the need to amplify the target region. Because the read lengths are limited in size, even small variations in amplicon lengths can bias the read counts toward smaller amplicons. Additionally, it is impossible to analyze unexpected gross chromosomal aberrations using targeted NGS.

Compared to other current CRISPR analysis programs that first align reads to a reference genome or sequence, CRIS.py is limited due to its strict text based search for flanking sequences. An unexpected SNP or indel present in either of the chosen flanking sequences will prevent proper analysis of the reads. It should be noted that the quality control measures employed in CRIS.py do flag these errors and alert the user to pick new flanking sequences. On the other hand, the strict sequence requirement can provide an advantage, making it is possible to incorporate SNP containing sequences into the program and examine editing events on specific alleles.

Overall, CRIS.py is a NGS analysis program that gives even novice users the ultimate flexibility and utility when analyzing one to thousands of edited pooled or clonal samples. Indeed, we are unaware of any upper limit to the number of samples that can be analyzed with CRIS.py, and have successfully analyzed over 16,000 fastq files with over a 100 test_sequences in one iteration of the script. CRIS.py outputs two editable files regardless of sample size, and results are presented in a format that is easily searchable and rankable enabling users to quickly identify successfully targeted clones. CRIS.py is nuclease platform agnostic and can be used to analyze genome editing outcomes using different genome engineering platforms including ZFNs, TALENs, and even CRISPR-Cas9-guided base editors. CRIS.py runs locally on most common computers and analyzes results in a matter of seconds to minutes. To our knowledge, there is no other NGS program for analyzing genome editing events that allows the direct comparison of multiple fastq files (samples) for multiple editing events.

## Supplementary information

Supplementary Figures

## Data Availability

Project name: CRIS.py. Project home page: https://github.com/patrickc01/CRIS.py. Operating systems: Platform independent. Minimum Hardware Requirements: Intel Core i3 with 2 or more cores and 2 GB RAM. Programming language: Python. Other requirements: Python 2.7, pandas library. Tutorial video can be accessed at the link below. https://s.stjude.org/video/player.html?videoId=6000021936001.

## References

[1] Brinkman, E. K., Chen, T., Amendola, M. & Van Steensel, B. Easy quantitative assessment of genome editing by sequence trace decomposition. *Nucleic Acids Res*, 10.1093/nar/gku936 (2014). 10.1093/nar/gku936 PMC4267669 25300484

[2] Hendel, A., Fine, E. J., Bao, G. & Porteus, M. H. Quantifying on- and off-target genome editing. *Trends in Biotechnology*, 10.1016/j.tibtech.2014.12.001 (2015). 10.1016/j.tibtech.2014.12.001 PMC4308725 25595557

[3] Sentmanat, M. F., Peters, S. T., Florian, C. P., Connelly, J. P. & Pruett-Miller, S. M. A Survey of Validation Strategies for CRISPR-Cas9 Editing. *Sci. Rep*. **8** (2018). 10.1038/s41598-018-19441-8 PMC5772360 29343825

[4] Pinello L (2016). Analyzing CRISPR genome-editing experiments with CRISPResso. Nat. Biotechnol..

[5] Güell M, Yang L, Church GM (2014). Genome editing assessment using CRISPR Genome Analyzer (CRISPR-GA). Bioinformatics.

[6] Park J, Lim K, Kim J-S, Bae S (2017). Cas-analyzer: an online tool for assessing genome editing results using NGS data. Bioinformatics.

[7] Wang X (2017). CRISPR-DAV: CRISPR NGS data analysis and visualization pipeline. Bioinformatics.

[8] Schirmer, M., D’Amore, R., Ijaz, U. Z., Hall, N. & Quince, C. Illumina error profiles: Resolving fine-scale variation in metagenomic sequencing data. *BMC Bioinformatics*, 10.1186/s12859-016-0976-y (2016). 10.1186/s12859-016-0976-y PMC4787001 26968756

[9] Schirmer, M. *et al*. Insight into biases and sequencing errors for amplicon sequencing with the Illumina MiSeq platform. *Nucleic Acids Res*, 10.1093/nar/gku1341 (2015). 10.1093/nar/gku1341 PMC4381044 25586220

[10] Cruaud, P., Rasplus, J. Y., Rodriguez, L. J. & Cruaud, A. High-throughput sequencing of multiple amplicons for barcoding and integrative taxonomy. *Sci. Rep*, 10.1038/srep41948 (2017). 10.1038/srep41948 PMC5292727 28165046

[11] Herbold, C. W. *et al*. A flexible and economical barcoding approach for highly multiplexed amplicon sequencing of diverse target genes. *Front. Microbiol*, 10.3389/fmicb.2015.00731 (2015). 10.3389/fmicb.2015.00731 PMC4503924 26236305

[12] Gohl, D. *et al*. An optimized protocol for high-throughput amplicon-based microbiome profiling. *Protoc. Exch*, 10.1038/protex.2016.030 (2016).

[13] U’Ren, J. M. & Arnold, A. E. Illumina MiSeq Dual-barcoded Two-step PCR Amplicon Sequencing Protocol. *protocol.io*, 10.17504/protocols.io.fs9bnh6 (2017).

