# Supplementary material for: CRIS.py: A Versatile and High-throughput Analysis Program for CRISPR-based Genome Editing
Source: Sci Rep. 2019 Mar 12;9:4194. doi: 10.1038/s41598-019-40896-w (PMC6414496; doi:10.1038/s41598-019-40896-w)
Supplement: Supplementary file 1 — Supplementary Figures [file 41598_2019_40896_MOESM1_ESM.pdf]

CRIS.py: A Versatile and High-throughput Analysis Program for CRISPR-based Genome Editing

Jon P. Connelly[1, 2] and Shondra M. Pruett-Miller[1, 2]

Supplemental Figure 1

a

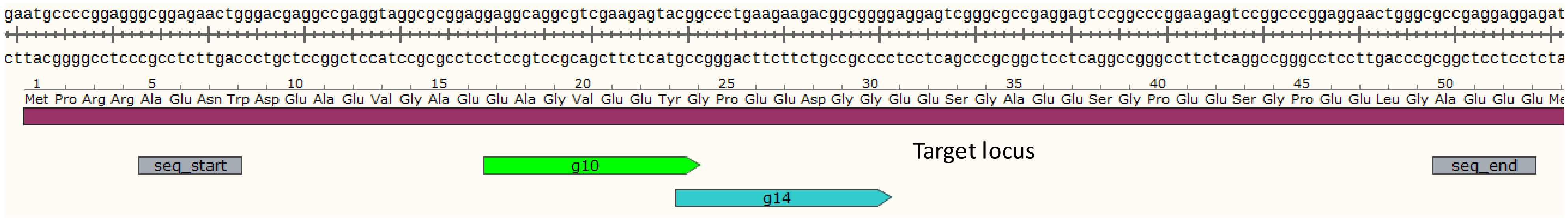

b

```
def get_parameters():
    #Note to user- Change text inside of quote marks '' for your experiment
  ①ID = 'Locus_1'
  ②ref_seq = str.upper('agggaatgccccggagggcggagaactgggacgaggccgaggtaggcgcggaggaggcaggcgtcgaagagtacggccctgaagaagacggcggggaggagtcgggcgccgaggagtccggcccggaagagtccggcccggaggaactgggcgccgaggaggagatgg')
  ③seq_start = str.upper('GCGGAGAACTG')
  ④seq_end = str.upper('GCCGAGGAGGA')
  ⑤fastq_files = '*.fastq'
  ⑥test_list = [
               str('g10'),   str.upper('GAGGCAGGCGTCGAAGAGTACGG'),
               str('g14'),   str.upper('CGGCCCTGAAGAAGACGGCGGGG')]

    return ID,ref_seq,seq_start,seq_end,fastq_files,test_list
```

Supplemental Figure 1. Example of parameters to run CRIS.py. **a,** Layout of example target locus with gRNAs (g10 and g14), seq_start, and seq_end labeled. **b,** The CRIS.py main file is edited and parameters (1-6) specific to each project are inputted by the user (text in red). "ID" is the name of the project. "ref_seq" is the expected WT reference amplicon. "seq_start" is a 10-20bp sequence found upstream of the gRNA target site. "seq_end" is a 10-20bp sequence found downstream of the gRNA target site. "fastq_files" are the fastq files you want to search. (Typically leave this as *.fastq). "test_list" contains the names and sequences which should be searched for.

Supplemental Figure 2

Name	Sample	Total	g1	g2	Total_indel	#1-Indel	#1-Reads(%)	#2-Indel	#2-Reads(%)	#3-Indel	#3-Reads(%)	#4-Indel	#4-Reads(%)
Miller-Plate37-A09	2 alleles	3814	11 (0.3%)	0 (0.0%)	100.00%	-10	2061 (54.0%)	-1	1743 (45.7%)	-11	6 (0.2%)	-2	4 (0.1%)
Miller-Plate38-B06	2 alleles	3916	4 (0.1%)	0 (0.0%)	100.00%	-5	2147 (54.8%)	-1	1756 (44.8%)	-6	6 (0.2%)	-2	6 (0.2%)
Miller-Plate38-F09	2 alleles	1165	4 (0.3%)	0 (0.0%)	100.00%	-1	662 (56.8%)	-11	498 (42.7%)	-2	4 (0.3%)	-18	1 (0.1%)
Miller-Plate37-B04	3 alleles	6041	2007 (33.2%)	0 (0.0%)	100.00%	-1	3945 (65.3%)	-2	2085 (34.5%)	-3	8 (0.1%)	0	2 (0.0%)
Miller-Plate37-F06	3 alleles	337	0 (0.0%)	0 (0.0%)	100.00%	-1	214 (63.5%)	-7	119 (35.3%)	-2	3 (0.9%)	-8	1 (0.3%)
Miller-Plate38-A10	3 alleles	2879	1 (0.0%)	0 (0.0%)	100.00%	-11	1911 (66.4%)	-2	957 (33.2%)	-12	5 (0.2%)	-3	2 (0.1%)
Miller-Plate38-B10	3 alleles	2655	8 (0.3%)	0 (0.0%)	100.00%	-1	1743 (65.6%)	-10	903 (34.0%)	-11	5 (0.2%)	-2	4 (0.2%)
Miller-Plate37-F11	3 alleles	3981	10 (0.3%)	0 (0.0%)	62.90%	0	1476 (37.1%)	-1	1306 (32.8%)	-4	1188 (29.8%)	-2	6 (0.2%)
Miller-Plate38-B08	3 alleles	4750	0 (0.0%)	0 (0.0%)	100.00%	-2	1626 (34.2%)	-8	1596 (33.6%)	-4	1496 (31.5%)	-5	13 (0.3%)
Miller-Plate37-C02	3 alleles	6916	2069 (29.9%)	2 (0.0%)	99.80%	-18	2418 (35.0%)	-8	2307 (33.4%)	1	2144 (31.0%)	0	16 (0.2%)
Miller-Plate37-B09	3 alleles	4725	9 (0.2%)	0 (0.0%)	100.00%	-1	1742 (36.9%)	-4	1535 (32.5%)	-2	1423 (30.1%)	-3	13 (0.3%)

Supplemental Figure 2. CRIS.py analysis allows copy number prediction in clonal populations. CRIS.py was used to screen single cell derived clones. The number of alleles in each clonal population can be predicted by the ratios of unique indels. Clones predicted to be triploid or diploid for the target locus based on indel fractions are highlighted in yellow or green, respectively.

Supplemental Figure 3

a

Test_Sequences:
CC: cttgaCCaatagccttgacaagg
TT: cttgaTTaatagccttgacaagg
TC: cttgaTCaatagccttgacaagg
CT: cttgaCTaatagccttgacaagg

Name	Sample	Total	CC	TT	TC	CT	Total_indel	#1-Indel	#1-Reads(%)	#2-Indel	#2-Reads(%)
Miller-Plate28-A01	Neg Control	861	831 (96.5%)	0 (0.0%)	0 (0.0%)	2 (0.2%)	0.50%	0	857 (99.5%)	-1	4 (0.5%)
Miller-Plate28-A03	Condition 1	978	519 (53.1%)	375 (38.3%)	21 (2.1%)	3 (0.3%)	0.70%	0	971 (99.3%)	-1	7 (0.7%)
Miller-Plate28-A05	Condition 2	797	456 (57.2%)	292 (36.6%)	13 (1.6%)	2 (0.3%)	0.80%	0	791 (99.2%)	-1	6 (0.8%)
Miller-Plate28-A06	Condition 3	944	479 (50.7%)	370 (39.2%)	32 (3.4%)	1 (0.1%)	1.10%	0	934 (98.9%)	-1	8 (0.8%)
Miller-Plate28-A09	Condition 4	1046	728 (69.6%)	278 (26.6%)	5 (0.5%)	5 (0.5%)	0.50%	0	1041 (99.5%)	-1	5 (0.5%)
Miller-Plate28-A10	Condition 5	954	670 (70.2%)	252 (26.4%)	7 (0.7%)	0 (0.0%)	0.30%	0	951 (99.7%)	-1	3 (0.3%)

b

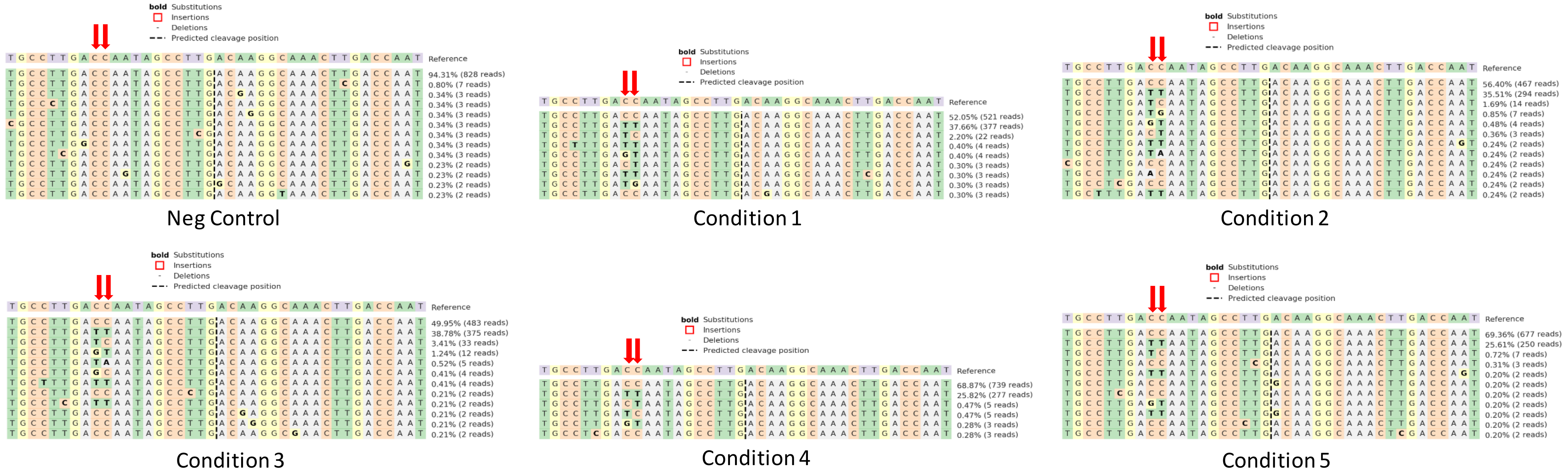

c

	CRIS.py				CRISPresso			
Sample	CC	TT	TC	CT	CC	TT	TC	CT
Neg Control	96.5%	0.0%	0.0%	0.2%	94.31%	0.00%	0.00%	0.00%
Condition 1	53.1%	38.3%	2.1%	0.3%	52.05%	37.66%	2.20%	0.00%
Condition 2	57.2%	36.6%	1.6%	0.3%	56.40%	35.51%	1.69%	0.36%
Condition 3	50.7%	39.2%	3.4%	0.1%	49.95%	38.78%	3.41%	0.00%
Condition 4	69.6%	26.6%	0.5%	0.5%	68.87%	25.82%	0.47%	0.47%
Condition 5	70.2%	26.4%	0.7%	0.0%	69.36%	25.61%	0.72%	0.00%

Supplemental Figure 3. CRIS.py and CRISPresso analysis of samples treated with BE4 base-editing reagents. a, Test_sequences and master summary file results from CRIS.py. **b,** Sequence alignment output from CRISPresso. **c,** Comparison of base-editing outcome frequencies obtained using CRIS.py or CRISPresso analysis. Only cytosine to thymine modifications within the target base-editing window are compared as marked by red arrows.

Supplemental Figure 4

a

$$\text{SNP test} = \frac{\text{total read count of seq\_start}}{\text{total read count of seq\_end}}$$

b

No SNPs in flanking sequences

```
seq_start                                   test_seq                                                                 seq_end
Allele 1   GACGGACTCTTGCTTTGTCGCCCAGGCTGGAGTGCAGTGGCGCAATCTCGGCTCACTGCAACCTCTGCTTCCTGGGTTCAAGCAATTCTCCTGACTCCGCCTCCCGAGTAGCTGGAATTACAGGCACCCGCCACCACACCTGG
Allele 2   GACGGACTCTTGCTTTGTCGCCCAGGCTGGAGTGCAGTGGCGCAATCTCGGCTCACTGCAACCTCTGCTTCCTGGGTTCAAGCAATTCTCCTGACTCCGCCTCCCGAGTAGCTGGAATTACAGGCACCCGCCACCACACCTGG
             seq_start count= 2                                                                                                        seq_end count=2

    seq_start (GGACTCTTGCT) total count = 2
    seq_end (CGCCACCACAC) total count = 2
      SNP test = 2/2 = 1
```

c

SNP in seq_start flanking sequence

```
seq_start                                   test_seq                                                                 seq_end
Allele 1   GACGGACTCTTGCTTTGTCGCCCAGGCTGGAGTGCAGTGGCGCAATCTCGGCTCACTGCAACCTCTGCTTCCTGGGTTCAAGCAATTCTCCTGACTCCGCCTCCCGAGTAGCTGGAATTACAGGCACCCGCCACCACACCTGG
Allele 2   GACGGACTCATGCTTTGTCGCCCAGGCTGGAGTGCAGTGGCGCAATCTCGGCTCACTGCAACCTCTGCTTCCTGGGTTCAAGCAATTCTCCTGACTCCGCCTCCCGAGTAGCTGGAATTACAGGCACCCGCCACCACACCTGG
            seq_start count= 1                                                                                                       seq_end count= 2

    seq_start (GGACTCTTGCT) total count = 1
    seq_end (CGCCACCACAC) total count = 2
      SNP test = 1/2 = 0.5
```

Supplemental Figure 4. SNP test. The SNP test is a QC measure to test if a fastq file contains reads with a SNP in either the seq_start or seq_end. **a,** During the SNP test, all reads are analyzed in the fastq file, and the total counts of seq_start is divided by the total counts of seq_end. **b,** If no SNPs are present in seq_start or seq_end, the ratio of reads between the two will be approximately 1. **c,** If a SNP is present in seq_start or seq_end, the total count of reads between seq_start and seq_end will differ by approximately two and give values of approximately 0.5 or 2 if the SNP is in seq_start or seq_end, respectively. It is recommended that SNP test results of .8 to 1.2 should be analyzed further and the start_seq and/or end_seq should be adjusted accordingly.

Supplemental Figure 5

a

$$\text{Raw\_wt\_test} = \frac{\text{Total read count of first test\_seq in fastq file}}{\text{Total read count of first test\_seq in seq\_match reads}}$$

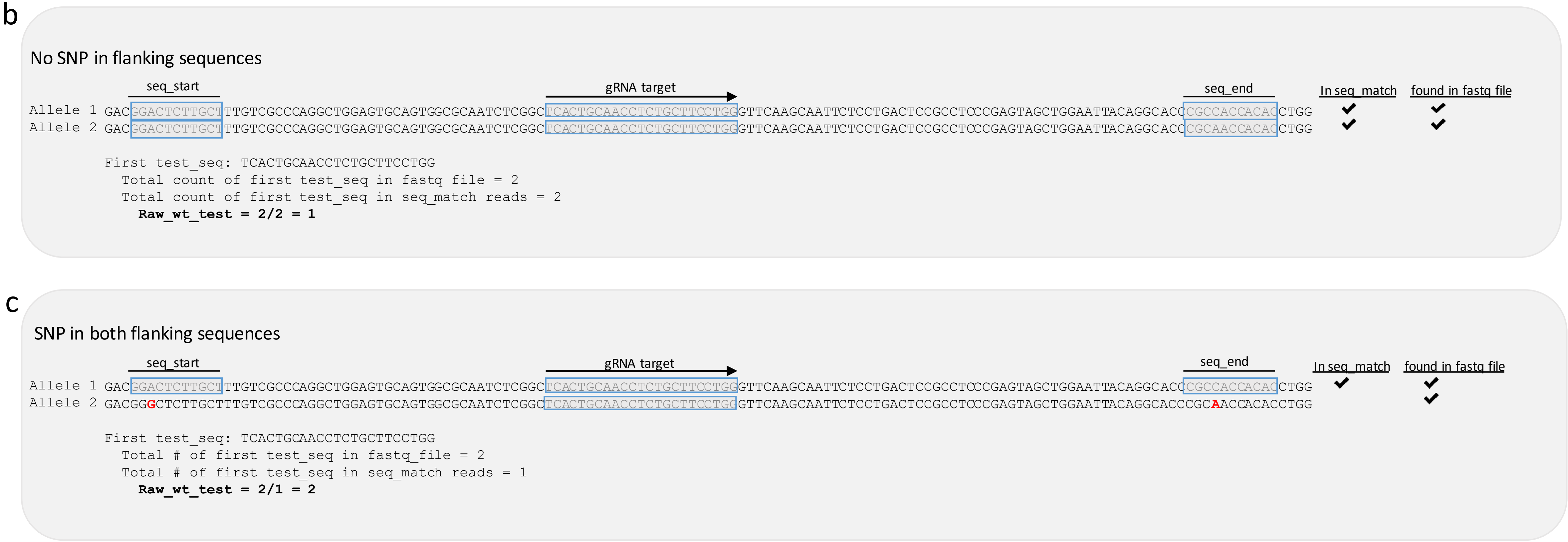

Supplemental Figure 5. Raw_wt_test. The Raw_wt_test is a QC measure to test if a fastq file contains reads with concomitant SNPs in both the seq_start and seq_end. **a,** During the Raw_wt_test, all reads are analyzed in the fastq file, and the total number of times the first test_seq is found in the raw fastq file (independent of seq_start and seq_end) is counted. Next, the total number of times the first test_seq is present in the seq_match sequences (containing seq_start and seq_end) are counted. The Raw_wt_test value is the ratio of these two counts. **b,** If no SNPs are present in seq_start and seq_end, the ratio of reads between the two will be approximately 1. **c,** If a SNP is present in seq_start and seq_end, then the total count of reads between seq_start and seq_end will differ by approximately two. It is recommended that Raw_wt_test results over 1.3 should be analyzed further and the start_seq and end_seq should be adjusted accordingly.
